# Cord Blood Cells for Developmental Toxicology and Environmental Health

**DOI:** 10.3389/fpubh.2015.00265

**Published:** 2015-12-03

**Authors:** Dora Il’yasova, Noreen Kloc, Alexander Kinev

**Affiliations:** ^1^Division of Epidemiology and Biostatistics, School of Public Health, Georgia State University, Atlanta, GA, USA; ^2^Creative Scientist, Inc., Durham, NC, USA

**Keywords:** developmental toxicity, cord blood cells, *in vitro* models, risk assessment, individual variability, high-throughput testing, endothelial progenitor cells, endothelial colony-forming cells

## Abstract

The Tox21 program initiated a shift in toxicology toward *in vitro* testing with a focus on the biological mechanisms responsible for toxicological response. We discuss the applications of these initiatives to developmental toxicology. Specifically, we briefly review current approaches that are widely used in developmental toxicology to demonstrate the gap in relevance to human populations. An important aspect of human relevance is the wide variability of cellular responses to toxicants. We discuss how this gap can be addressed by using cells isolated from umbilical cord blood, an entirely non-invasive source of fetal/newborn cells. Extension of toxicological testing to collections of human fetal/newborn cells would be useful for better understanding the effect of toxicants on fetal development in human populations. By presenting this perspective, we aim to initiate a discussion about the use of cord blood donor-specific cells to capture the variability of cellular toxicological responses during this vulnerable stage of human development.

## Introduction

Blood from umbilical cord (cord blood) has been recognized as a source of hematopoietic cells useful for transplantation to patients with malignant or genetic diseases (1). At the same time, cord blood is a source of a fetal/newborn material that is being obtained in an entirely non-invasive way. Cord blood contains stem and progenitor cells, which are possible to isolate, expand *in vitro*, and cryopreserve. A library of cord blood-derived stem/progenitor cells can represent at a cellular level a population of newborns. Such a library could be a powerful tool to study the variability of human fetal responses to a wide variety of developmental toxicants. Studying individual variability is rooted in an epidemiological approach that is not currently incorporated into standard toxicological testing. Use of cord blood cells allows for direct toxicological testing in the most vulnerable human populations (i.e., human fetuses and newborns), and thus, overcomes limitations associated with traditional *in vivo* and *in vitro* test methods.

## Use of Animal Models in Prediction of Human Responses to Environmental Hazards

Developmental toxicology focuses on predicting the ability of environmental hazards to cause anatomical and functional birth defects in humans (2). Currently, developmental toxicology depends on studies employing *in vivo* animal models supported by data derived from molecular and cell-based models (3, 4); the *in vivo* models employ pregnant animals, primarily, rats, and rabbits (3). Such models allow observing pharmacokinetics and pharmacodynamics with the main outcome being an incidence of malformations. This information provides the main basis for public health decisions regarding safe levels of exposure to drugs and toxicants, including pesticides and industrial chemicals (3, 5). Specifically, the Food and Drug Administration (FDA) according to the International Conference on Harmonization (ICH) S5(R2) document mandates new pharmaceutical agents to be tested for developmental toxicity in two animal species (6, 7). The assumptions used in regulatory practice consider humans as more sensitive than animals; if a compound is developmentally toxic in animals, it is assumed to be toxic in humans at blood levels that are within 20-fold of the therapeutic blood level (5). The high cost and inefficiency of the *in vivo* animal testing have been recognized; these limitations can be partially mitigated by using appropriate *in vitro* models (8–13).

Widely accepted today, *in vitro* testing includes the embryonic stem cell test for embryotoxicity, the micromass embryotoxicity assay, and the whole rat embryo embryotoxicity assay (8–13). These test methods are used to assess the toxicity of a substance or side effects of a drug on embryonic cells while avoiding the influence of maternal toxicity (14). The embryonic stem cell test is performed using a mouse embryonic stem cell line (mESC) and differentiated 3T3 fibroblasts. Inhibition of mESC differentiation into cardiomyocytes serves as an indicator of developmental toxicity, while increased cytotoxicity in mESC vs. fibroblasts provides information on the specificity of toxicity to embryonic development. The micromass embryotoxicity assay utilizes embryonic cells isolated from a rat embryo. Most frequently, this assay uses cells from developing limb buds or midbrain to approximate how compounds influence organ growth over a limited time period of embryonic development. The whole rat embryo assay assesses signs of malformation or retardation in cultured whole embryos. A combination of these approaches was used to test 20 therapeutic drugs, 16 of them were contraindicated in pregnancy and 4 drugs that were considered to be safe (15). This study used P19C5 mouse embryoid cells and NIH/3T3 fibroblasts (15). P19C5 mouse embryoid cells exhibit axial elongation morphogenesis *in vitro* and, therefore, were used not only as a cellular representation of an embryo but also as a model of embryonic growth, differentiation, and morphogenesis. The tested drugs presented a wide spectrum of therapeutic targets and chemical properties, including acitretin (treats skin diseases), diethylstilbestrol (prevents miscarriages and premature deliveries), doxylamine (anti-histamine for allergies), lovastatin (reduces LDL cholesterol), and others. This study showed a strong correlation between the *in vitro* observed effects and the expected developmental toxicity of the drugs (15). Thus, the described approaches cover a variety of toxicological effects from cellular toxicity to organ formation and growth retardation.

The unique challenge to studying developmental toxicology in mammals (including humans) is the lack of accessibility to the developing fetus. This is addressed to a limited extent by non-mammalian animal models that use amphibians, avians, or fish and allow direct observation of how different concentrations of a compound influence embryonic development. The major advantage of these models is the ability to observe toxicological effects on spatial and temporal development of the whole animal. For example, amphibian metamorphosis depends on thyroid hormone function along the hypothalamic–pituitary–thyroid axis (16). The amphibian tadpole bioassay evaluates a number of morphological endpoints, including thyroid gland histopathology, hind limb and snout-vent length, and wet body weight. The molecular aspect of this assay identifies disruption of thyroid function, including perturbations in thyroid hormone synthesis, transport, and metabolism by environmental contaminants (16). Widely recognized and included in the Organization for Economic Co-operation and Development (OECD) and the Environmental Protection Agency (EPA) guidelines, African clawed frog (*Xenopus laevis*) metamorphosis is being used for screening potential endocrine disruptors (17). The new sub-specialties using whole organisms include functional toxicology or toxicogenomics based on the models with defined genetic background; such models allow for assessing the role of specific gene products in tolerance to chemicals during embryonic development (18). Specifically, organisms harboring gene deletions or depleted proteins are used to examine genetic requirements for toxicity tolerance. In this regard, zebrafish is a highly promising model offering several advantages. A transparent chorion and embryo allow for direct observation of morphological development. Furthermore, these observations can be connected to a complex behavior in the adult zebrafish due to a short maturation period, which provides a window to developmental neurobehavioral testing (19). Together, these tests provide valuable information on how toxic a specific compound can be to embryonic development.

To what extent are these models applicable to human embryonic and fetal development? This question remains one of the major challenges of current developmental toxicological testing. It appears that a fundamental gap between animal – both *in vivo* and *in vitro* – models and an assessment of human health risks lies in the fact that developmental responses to compounds are influenced by the reproductive strategies of the species (20). Short-lived species with rapid reproduction and long-lived species with few offspring and delayed maturation represent two extremes. In that respect, short-lived rodents can model human development only to a limited extent (21). Non-mammalian models are likely to be even less relevant to human development. We contend that this gap in whole organism models can be breached by using *in vitro* data. Taking into account that fundamental cellular responses are conserved across the species, *in vitro* embryotoxicity models remain an important research tool, and can become part of regulatory decisions, given that the results are supported by the available human data. In this context, utilizing cord blood cells may prove to be valuable in advancing the assessment of developmental toxicological effects as the cells are obtained from the umbilical cord of a human at the boundary of fetal and newborn development.

## Cord Blood as a Source of Material for Developmental Research

**Figure 1:**
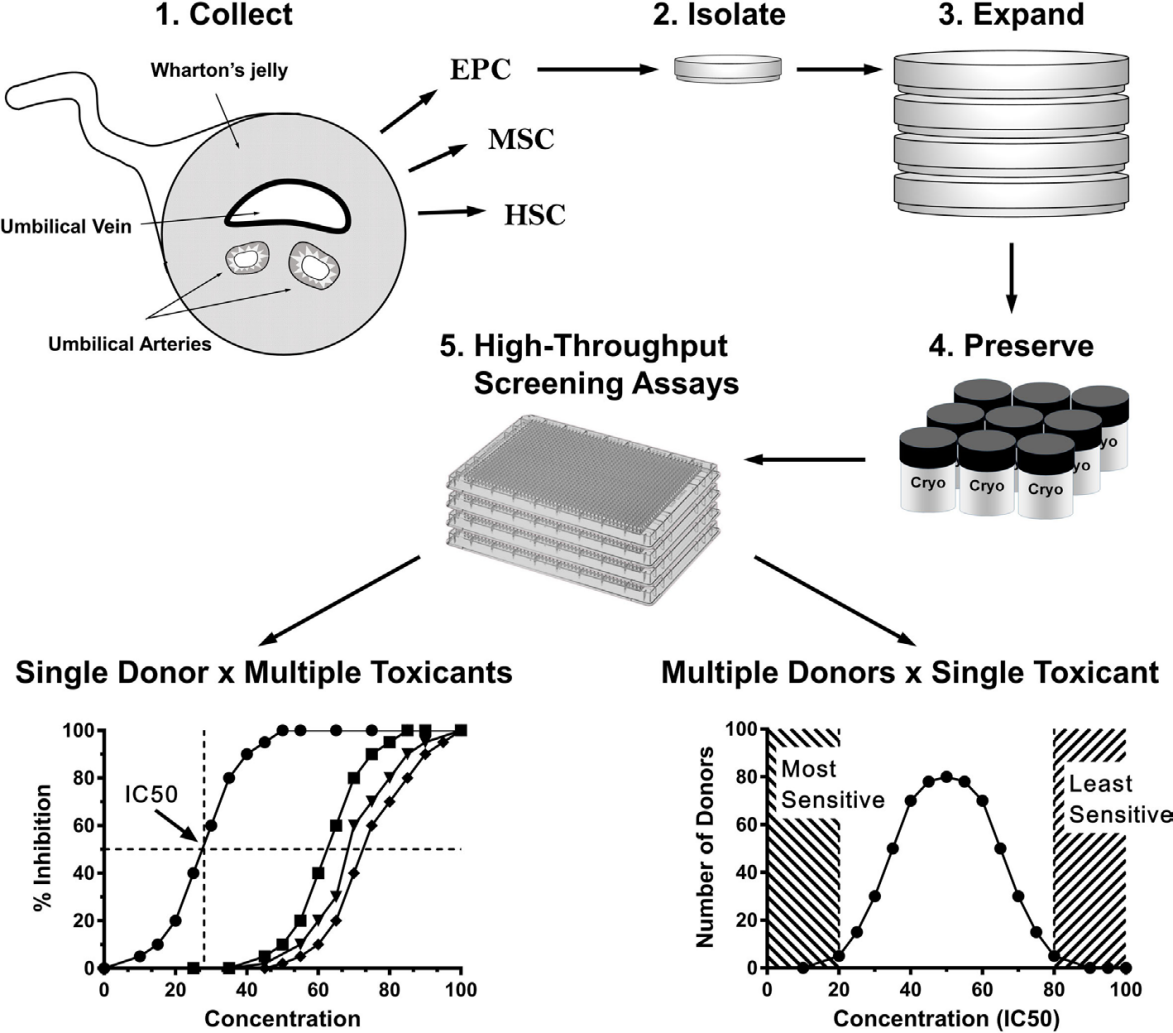
**Use of newborn stem/progenitor cells for assessment of individual and/or population-wide sensitivity to toxicants**. Steps 1–4: a library of donor-specific cells can be used in a variety of high-throughput screening (HTS) assays depicted in Step 5. HTS assays provide information for either personal (lower left chart) or population-wide (lower right chart) toxicological assessment. Left chart shows dose-dependent inhibition of a cellular function (such as proliferation). Based on this curve, a chemical dose leading to 50% inhibition of a cellular function IC_50_ is determined. Right chart shows a hypothetical distribution of cellular responses (IC_50_) to a given toxicant. Dashed areas represent arbitrary thresholds for both most sensitive (left) and most resistant (right) phenotypes (individuals). HSCs, hematopoietic stem cells; MSCs, mesenchymal stem cells; EPCs, endothelial progenitor cells.

The umbilical cord connects fetal circulation to the placenta providing nutrition and oxygen to the embryo while removing metabolic waste. It consists of two arteries and one vein surrounded by a mucoid connective tissue called Wharton’s jelly. Both Wharton’s jelly and the blood (cord blood) present a rich source of fetal material, including stem and progenitor cells (Figure 1) (22–27). The umbilical cord is a unique organ because it belongs to both fetus and newborn. Cord blood cells are formed during fetal development and harvested at birth. Fetal hematopoiesis presents a complex process that occurs in different anatomical locations, depending on the stage of gestational development (28, 29). It appears that cord blood cells are generated through expansion and maturation of hematopoietic stem cells (HSCs) from very early developmental stages. Essentially, cord blood presents a non-invasive source of fetal/newborn tissue.

Therapeutic potential of cell-based treatments have stimulated intensive research efforts on how to isolate, expand, and preserve various cell types from cord blood. In particular, cord blood is a source of HSCs, which have considerable therapeutic value because of their blood-forming capacity and ability to re-populate bone marrow in patients affected by cancer or other blood and immune system disorders (30). Like other stem cells, HSCs slowly divide *in vivo* and hardly proliferate *in vitro* (31, 32). Currently, proliferation of HSCs *ex vivo* presents an area of very intense research effort in cord blood transplantation because a low number of proliferative blood-forming cells limit successful restoration of the recipient’s bone marrow (24, 33). The use of HSCs for other purposes, such as developmental research or epidemiological studies, is also limited waiting for the development of reliable methods of cell culture expansion and cryopreservation.

A variety of phenotypically distinct (non-hematopoietic) precursor cells, apart from HSCs, can be isolated from cord blood and/or Wharton’s jelly (22, 23). Some of these cells have considerable proliferation potential *in vitro* and, thus, can be employed for both therapeutics and developmental research. For example, mesenchymal stem (stromal) cells (MSCs) can be isolated from cord blood and produce highly proliferative cultures (34, 35). With an appropriate stimulation, MSCs differentiate into a variety of tissues, including bone, cartilage, fat, or muscle (34, 36–38). Importantly, MSCs lack immunogenic cell surface markers, which are critical, for the success of transplantation. Therefore, *in vitro* expanded and differentiated MSCs can be potentially very useful for regenerative medicine, for example, to improve heart function after myocardial infarction (39). However, the rate of MSCs isolation from cord blood is currently <50% (34, 35). This means that MSCs can be isolated from only a subset of newborns. It remains unknown, what defines such selectively and whether it is important for transplantation purposes. Likewise, population-wide studies involving MSCs, although on the horizon, do not seem feasible at the moment.

For population-wide research, endothelial progenitor cells (EPCs) represent a better choice. EPCs are easy to isolate, expand *ex vivo*, and cryopreserve. EPCs are circulating cells capable of proliferating and differentiating into mature endothelial cells (40, 41). Identification of putative circulating EPCs that are involved in postnatal vasculogenesis and vascular repair was reported in 1997 (42, 43). EPCs are characterized by expression of surface markers CD34 and VEGFR-2 (KDR and CD309) and the ability to uptake acetylated-LDL and to bind *Ulex* lectin. Subsequent studies have revealed that true EPCs do not express the leukocyte common antigen CD45 and form colonies that appear after 2–3 weeks in culture producing long-term highly proliferative donor-specific lines (44, 45). These cells were named late outgrowth endothelial cells or endothelial colony-forming cells (ECFCs). Because of their involvement in neovascularization, ECFCs are being studied as a therapy for ischemic limb or heart disease and as an imaging probe for vasculogenesis (46). From our own experience, ECFCs isolated from cord blood are especially interesting for developmental studies because of the high success rate (~90%) of their isolation. We routinely isolate ECFCs from cord blood and use them to study individual responses to drugs, chemical toxicants, and other environmental hazards. Particularly, we have found that low doses of ionizing radiation affect their ability to proliferate (47); the magnitude of the effect appears to be donor specific. The direct measurement of cell proliferation presents an example of how donor-specific cells can be used to rank individuals by cellular sensitivity to radiation; such ranking can hardly be inferred from the genomic, epigenomic, metabolomics, or transcriptional profile data. Using a similar approach, we received preliminary data indicating that the proliferation of ECFCs can be also inhibited by exposure to chemical toxicants, i.e., endocrine disruptor Bisphenol A and heavy metal cadmium.

In summary, cord blood cells represent a rich source of fetal/newborn cells. The limiting factor in using HSCs and MSCs for studying individual variability of cellular responses lies in lack of the effective techniques allowing for their isolation and expansion *in vitro*. By contrast, such techniques exist for ECFCs. Theoretically, a collection of donor-specific ECFCs can serve as a model for assessing individual variability in response to toxicants, because ECFCs represent cells in circulation that are in direct contact with ingested/absorbed toxicants and their metabolites. Expansion of this approach to MSCs, other cell types differentiated from MSCs, and HSCs can be realistic in the near future as the methodology for their isolation is rapidly developing.

## Cord Blood Cells Present an Opportunity to Advance Developmental Toxicology

As a potential platform for fetal toxicity testing, cord blood-derived MSCs, ECFCs, and other progenitor cells have several advantages. As noted above, these cells provide an opportunity to directly test how environmental toxicants affect the dynamics of cellular function and metabolism, arising from a complex interaction between genes, proteins, and cellular organelles. Donor-specific cells carry not only the entire genomic but also possibly the epigenomic information of a donor. We suggest that a collection of donor-specific cells can potentially capture a spectrum of human variability in response to a certain toxicant or a class of toxicants (Figure 1).

Unlike epidemiological focus on individual variability within the population, the traditional toxicology testing does not address this aspect of environmental interactions but aims to answer the question of whether a specific agent is or is not toxic at a specific dose (48). The traditional toxicological testing minimizes variability of results by using a standardized test method that reduces the inherent variability of a biological model (e.g., using an inbred rodent strain). This contrast between standardization and the biological diversity of human populations has not been addressed. Extension of toxicological testing to collections of human fetal/newborn cells may be essential in order to better understand the effect of toxicants on fetal development in human populations. Using cord blood-derived stem and progenitor cells can address the following questions:

1. What is the lowest dose of a toxicant affecting human fetal cells? Is this dose different between cell types? Direct measurements of donor-specific cellular responses allow researchers to determine the range of fetal/newborn variability in response to toxicants. ECFCs, MSCs, and other cord blood-derived cells can give rise to a variety of cell types, including mature endothelial cells, osteoblasts, adipocytes, chondrocytes, neural, muscle, and myocardial cells. Variability of responses between cell types will provide information on tissue target.
2. Are fetal cells derived from cord blood more vulnerable to developmental toxicants as compared to human adult cells? To which degree are the results of cord blood cell testing concordant with animal-based *in vitro* and *in vivo* models? These proposed comparisons extend the existing framework of embryotoxicity testing and may provide valuable information on similarities and differences of cellular responses derived from animals and humans.
3. How similar are the cellular mechanisms responsible for toxicological effects in cord blood cells and animal *in vitro* models? Although cellular mechanisms are assumed to be conserved, the difference in toxicity between embryonal and adult cells is widely acknowledged. Extending this logic to inter-species variability, confirmation of mechanistic similarity in responses between embryonic mouse and human fetal cells provides additional in-depth validation of *in vitro* testing of developmental toxicants.
4. Can fetal cells be used to evaluate the teratogenic potential of chemical toxicants? In comparison to the classical animal model evaluating teratogens, all cellular models, including cord blood cells, have obvious limitations. *In vitro* testing of cellular responses cannot be extrapolated to teratogenic effects directly. However, this model can be used as the first line screening to evaluate teratogenic potential in comparison with the effects of known teratogens with a similar chemical structure. This approach has been used in evaluating developmental neurotoxicity of organophosphorus flame retardants (49).

These perspectives provide a focus for further discussions of the population-based approach in developmental toxicology. In the next section of this review, we discuss how this approach fits into the global shift within toxicology science.

## Donor-Specific Collections of Primary Cord Blood Cells Address the Shift in Toxicity Testing Approaches: Focus on High-Throughput Capacity and Biological Mechanisms

The National Research Council’s Committee on Toxicity Testing and Assessment of Environmental Agents produced the report “Toxicity Testing in the 21st Century: A Vision and a Strategy” outlining the need for improvements in evaluating human risk posed by the thousands of chemicals in use (50, 51). A collaboration known as Tox21 was formed including the National Institute of Environmental Health Sciences (NIEHS)/National Technology Program (NTP), the U.S. Environmental Protection Agency’s (EPA) National Center for Computational Toxicology (NCCT), the National Human Genome Research Institute (NHGRI)/National Institutes of Health (NIH) Chemical Genomics Center (NCGC) [now located at the National Center for Advancing Translational Science (NCATS)], and the U.S. FDA (51, 52). Through the Tox21 program, toxicology is shifting from a predominately observational science at the level of disease-specific models *in vivo* to a predominantly predictive science focused on inclusion of target-specific, mechanism-based, and biological observations *in vitro* (52). Such shift to *in vitro* testing is justified by its higher capacity and lower cost compared with *in vivo* models and the potential for data more directly relevant to humans.

### Next-Generation Toxicological Research Aims to Implement High-Throughput and Population-Wide Testing of Cellular Responses to Toxicological Agents

The EPA is required under the Toxic Substances Control Act (TSCA) to compile and publish a list of each chemical substance that is manufactured or processed in the U.S. that requires toxicological testing (53–55). Exempt from TSCA are drugs, cosmetics, foods, food additives, pesticides, and nuclear materials, which are regulated by other agencies (53, 54). Approximately 500–1,000 new substances are added to the TSCA inventory each year (53). Currently, there are over 84,000 chemicals in use with ~4,000 chemicals produced in high production volume (above 1 million pounds annually) (53, 54, 56). The Tox21 program has compiled a compound library of >10,000 substances of toxicological interest, including pharmaceuticals (51).

Addressing this problem of inadequate toxicological data on a large number of chemicals, Tox21 proposed cell-based *in vitro* assays as the first screen test for prioritizing substances for further toxicological evaluation (56–58). Cell-based assays can process >100,000 compounds per day making this high-throughput screening approach efficient in time and cost as well as allowing testing compounds at many concentrations (52, 57). The Tox21 program tests all compounds at typically 15 concentrations to generate concentration–response curves (51, 52). This dose–response-based method is highly reproducible generating significantly fewer false-positive and false-negative results as compared to the traditional approach used for drug discovery, which tests compounds at only one concentration (51, 52).

As articulated by Collins et al. (52), the focus on human relevance requires *in vitro* testing of human cell lines. As noted above, an important element of human relevance is population variability in responses to toxicological agents (59, 60). The recently published study by Abdo et al. (59) presents an example and sets up the methodological stage for a transition toward population-based *in vitro* testing. This study assessed variation in cytotoxic response to 179 chemicals in 1,083 lymphoblastoid cell lines (peripheral B lymphocytes immortalized with Epstein–Barr virus). The cell lines represent nine populations from five continents, including individuals of European (USA, Great Britain, and Italy), Chinese (China), Japanese (Japan), African (Kenya and Nigeria), Mexican (Los Angeles, CA, USA), and Colombian (Colombia) ancestries.

Abdo et al. (59) used EC_10_ (10th percentile; concentration for which cell viability was 90%) as the lowest concentration inducing a significant effect on cell growth. The variability of EC_10_ values across the cell lines was several orders of magnitude for the majority of chemicals. These results unequivocally demonstrate that individual variability in human responses to environmental toxicants cannot be ignored in risk assessment. Toxicological testing using human material (i.e., *in vitro* cell-based testing) represents the only option to assess such variability.

This study by Abdo et al. is remarkable in several ways. Lymphoblastoid cell lines have been criticized as a model, because these are transformed cells and because they represent only one highly specialized cell type. How can the results of such testing be extrapolated to other cell types and more importantly to humans *in vivo*? Despite these limitations, the study by Abdo et al. validated the concept that a cell can represent an individual at least to some degree. First, it demonstrated that inter-individual variability of the *in vitro* results is comparable to *in vivo* human toxicodynamic variability. Second, the study connected phenotypic biochemical testing with genetic variability, thereby setting up the example of how *in vitro* testing can be used for genome-wide association studies of human genetic susceptibility to chemicals. Specifically, the diversity in cellular responses was mapped to the genes with proteins that are localized to cell membranes, including solute carriers, which have been identified as potential mediators of antineoplastic drugs’ cytotoxicity (61, 62). Thus, the results derived from this testing of highly specialized (and immortalized) cells do inform on the general variability of individual response to a toxicological agent. As direct *in vivo* validation of human *in vitro* testing is impossible, these results set up examples of indirect validation using the existing human data.

Primary cell lines from cord blood cells fit ideally into this conceptual framework. We and others demonstrated that ECFCs have high proliferation potential *in vitro* and, thus, are suitable for high-throughput assays (47, 63, 64). As lymphoblastoid cell lines represent to a considerable extent the biological individuality of the donors, it is logical to expect that biobanks of primary ECFC cell lines from cord blood could represent populations of newborns. In addition, there is an advantage of using primary as opposed to transformed cell lines as primary cells provide an opportunity to test important cellular targets of developmental toxicology, such as cellular growth and differentiation. Taking into account that human fetuses and newborns represent vulnerable populations with respect to environmental toxicants such *in vitro* testing of cord blood cells may uncover a different range of EC_10_ as compared to adult cells.

### Focus on Mechanism-Based Biological Observations *In Vitro*

Focus on functional mechanism-based assays can be illustrated by high-throughput testing of mitochondrial function. Mitochondria have been recognized as a sensitive target for environmental compounds, because these organelles play a central role in cellular responses to environmental stressors (65). NCATS conducted the first high-throughput screening of a large number of chemicals to assess their effect on mitochondrial function. Attene-Ramos et al. assessed 1,340 unique environmental and drug-like compounds for their ability to change mitochondrial membrane potential in human hepatocellular carcinoma cells (HepG2) (58).

This approach identified 255 (~20%) compounds that decreased mitochondrial membrane potential in a concentration-dependent manner (58). Furthermore, the second round confirmatory testing used a different readout and showed a high concordance rate of >90% with the first line screening (58). In addition, seven of the inactive analogs were reevaluated with all confirming their inactive status (58). Compounds were further assessed for mechanism of action by measuring changes in oxygen consumption rate; this testing identified 20 compounds as uncouplers (inhibitors of the ATP synthesis via dissipation of membrane potential) (58). This comprehensive approach using primary quantitative high-throughput screening assays with in-depth secondary screening is an example of studying the mechanism of action (58). This test method was subsequently used to screen the Tox21 10,000 compound library (66).

Extension of this approach to primary cell lines isolated from cord blood may bring an unprecedented rate of discoveries that will help to reveal how exactly environmental toxicants influence early human development. A continuum of data delineating-specific biological mechanisms can connect developmental toxicology to human populations.

## Conclusion

Cord blood cells have been used for therapeutic purposes. At the same time, cord blood presents a non-invasive source of human fetal cells. Cord blood stem and progenitor cells can be isolated, expanded *in vitro*, and cryopreserved. Current techniques, which are rapidly expanding, allow for the isolation and expansion *in vitro* of ECFCs and MSCs. Whereas ECFCs can be easily isolated from cord blood, MSCs are currently isolated from ~50% of the specimens. Thus, the population-based approach is feasible currently only with ECFCs.

As the principles of toxicological testing are shifting toward the use of more *in vitro* models, a testing platform based on primary fetal cell lines derived from cord blood fits well into this research paradigm. Importantly, it has been demonstrated that human cell lines representing different individuals could capture a wide variability of individual responses to a tested toxicant. Similarly, a collection of primary cord blood-derived cell lines can inform developmental toxicology about the variability of fetal/newborn responses to a toxicant. As with any model, use of cord blood cells as a proxy of a human fetus/newborn has its limitations. *In vitro* testing of cellular responses cannot be directly extrapolated to many teratogenic effects, such as malformations or childhood obesity. However, this model can be used to evaluate a teratogenic potential in comparison with the effects of known teratogens of a similar chemical structure. With this review, we would like to open a discussion of the exciting opportunities of using cord blood stem and progenitor cells as a window into *in utero* and early postnatal development.

## Author Contributions

DI and AK developed the structure and defined the main content of the manuscript. All three authors wrote different parts of this review.

## Conflict of Interest Statement

Alexander Kinev is a founder and executive director of Creative Scientist, Inc. The remaining authors have no other conflict of interest to declare.
